# 

**Published:** 1801-12

**Authors:** 


### NEW MEDICAL PUBLICATIONS.

Hygeia, a Series of Essays on Health, on a Plan entirely popular. By THOMAS BEDDOES, M. D. (to be completed in Twelve or Sixteen Monthly Numbers,) 2s. Phillips.

A Treatise on the Cow-pox, containing the History of Vaccine Inoculation, and an Account of the various Publications which have appeared on that Subject in Great Britain, and other Parts of the World. By JOHN RING, Part I. 8s. boards. Johnson.

Cases of Phthisis Pulmonalis, successfully treated upon the Tonic Plan; with Introductory Observations. By C. PEARS.

The Modern Practice of Physic, 2 vol. 8vo. By ROBERT THOMAS, M. D.

---

### To CORRESPONDENTS.

*The unexampled influx of Original Communications this month, has obliged us to postpone the Account of English Books, and part of the Foreign Literature, as well as several Original Papers.*

*Communications are received from Drs. Moises, Noehden, and Veiuxseux; Messrs. Hull, Cooke, Observator, M. D. and D. T.*

---

### ERRATA.

Page 218, l. 20, to W. B. Bayley, *add* M. D.
Page 459, l. 20, *for* temperance, *read* temperature.
Page 451, l. 41, *for* salutary, *read* solitary.

---

END OF VOL. VI.

### INDEX.

Page.

ABILDGAARD, Prof. experiments by, to ascertain the quantity of carbon in the blood 192
——— on the oxydation of blood as the effect of respiration 373
Abscess, an extraordinary case of 293
Acetic Ether, the good effects of, in rheumatism and the gout 273
Acid, on the use of it in fever 87
—— experiments on the muriatic 475
—— on the internal use of phosphoric 92
Acids, Dr. Beddoes's plan for trying 105
Acoroides, or Botany-bay gum, uses of in complaints of the intestines 429
Acoustic tubes, examination of (with a plate) 75
Adams, Dr. on sending consumptive patients to Madeira 415
——— on aneurism 535
Address to the poor on vaccine inoculation 115
Ægypt, on the endemic ophthalmia of 357
Æthiop's antimonialis, a short and easy mode of preparing 15
Affusion cold, in fever, Dr. Rogers on 106
——— Dr. Currie on 381
Agust earth, description of, a new discovery so termed by Prof. Tromsdorff 473
Air, experiments on land and sea 203, 359
Anatomical remarks on the ornithorynchus paradoxus 73
Anatomical preparations, method of preserving 274
Ancylosis incompleta, relieved by warm oil 71
Aneurism, Dr. Adams's case of 535
Angina pectoris, two cases of 320
——— polyposa spasmodica, description of 94
Animal chemistry, remarks on 416
——— impregnation, on the proximate cause of 254
Animals, on the odours exhaled by living 339, 439
Animation, essay on suspended 116
——— suspended, review of Dr. Sturve's treatise on 8

Page.

Antimonium Tartarisatum, its use in preventing bilious fever 311
Apparatus, descriptive of the resuscitative 84
Apoplexy, Mr. Crowfoot's statement of a case of 549
——— answer to the same 552
Argillaceous earth, how analysed 373
Arnemann's, Prof. examination of acoustic tubes 75
Arsenic, on the use of in cancer 31
Arteries, on the cause of their being found empty after death 379
Arthritic pains, relieved by the vapours of the hepatic gas 92
Arthritis, description and cure of 454
Asthenic ulcers, cured by caustic 34
Atkinson's, Mr. account of an extraordinary cancer (with a plate) 448
Bacon, Lord, his merit as a discoverer 409
Barry, Dr. on vaccina 423
Parytes, description of and how analysed 374
——— method of preparing the muriated without kali 467
Bath, account of the diseases in the dispensary at 195, 364, 388, 491
Batty, Dr. his lectures announced 380
Bayley, Mr. on a schirrhous pancreas as a cause of the jaundice 218
Beddoes's, Dr. questions concerning the children of the poor 24
——— plan for trying acids 165
——— announcement of his popular essays 362
Bell's, Mr. system of dissections, reviewed 80
Belladonna, on the use of in hydrophobia 93
——— in extracting the cataract 352
——— in the scarlet fever 368
Benevolent Medical Society, resolutions of 113
Bennett's, Mr. case of an abscess 293
Berkeley's, Lord, letter to Dr. Jenner on the cow pox 105
Berthollet, Cit. on eudiometry 376
——— experiments by, on the muriatic acid 475

Page.

Bestucheff, method of preparing the nervous tincture of 474
Bichat, Cit. on the arteries after death 379
———— membranes 449
Bilious fever, on the prevention of, by antimonium tartarizatum 311
Birds, on the organ of the voice, of 279
Bishop, Mr on suspended animation 315
Black, Dr. on the chemical discoveries of 409
Blair, Mr. on œsophagotomy (with plate) 97
———— on cancerous hydatids 192
———— his proposals for a history of hospitals 303
———— his lectures announced 476
Blane's, Dr. communication on the ophthalmia of Ægypt 357
Bliss's, Mr. observations on the Kilburn waters 332
Blisters, their use in suppression of urine 526
Blood, experiments to ascertain the quantity of carbon in the 192
———— examination of the colouring of the 469
———— on the vitality of the 513
Blount's, Mr. letters on the cow pox 114
Blumenbach, Dr. on the ornithorynchus paradoxus 73
Bobba, Dr. on the cause of rickets 191
Boeckmann's, Mr. experiments on the influence of light on phosphorus 374
Bones, Prof. Isenflamm, on the marrow of the 470
Books, Medical, Critical Retrospect of
Wrisberg's medical treatises, 77. Dr. Posewitz's journal for medicine, 79. Prof. Wiedemann's archives for zoology and zootomy, ib. Bell's system of dissections, 80. Dr. Struve on suspended animation, 83. Dr. Reich on fever, 86 Reece's medical and chirurgical pharmacopœia, 88. Dr. Sutton on pulmonary consumption, 89. Dr. Hufeland's system of practical medicine, 170. Corvissart's &c. journal of medicine, 174. Le Febure's researches, &c 175. Prof. Hildebrand's pocket-book for health, *ib.* Dr. Thomson's family physician, 176. Dr. Nisbet's practical treatise on diet, 178. Geohegan on the venereal disease, 180. Dr. Kilian's genius of health and life, 253. Dr. Osiander's treatise on the cow pox, *ib.* Pulley on animal impregnation, 254 Dr. Wilson on febrile diseases, 255, 335.

Page.

Bouillon Lagranges, his method of preparing syrup of quicksilver 15
Boutflower, Mr. on opiate friction 433
Bouttatz, Dr. his account of an extraordinary tumour in the eye (with plates) 289
———— cases of catalepsy and epilepsy 425
———— question to, by Dr. Kinglake 461
Bradley, Dr. letter to, from Dr. Clarke 95
———— his lectures announced 380
Bristol, account of diseases in the dispensary at 4, 112, 196, 364, 388, 491
Brugnatelli, Prof. on the transition of tartarous, into saccharic acid 475
Brush, description of Molwitz's metallic (with a plate) 91
Buboes, on the use of snails in obstinate 93
———— description of the pestilential 260
Cachexy, description and method of treating of 376
Cæsarean operation, a case of, by Mr. Wood 346
Calcareous earth, how analysed 374
Calculus, account of an extraordinary (with a plate) 391
Cambridge (North America) meteorological register in the university of 528
Camphor, Mr. Ring on the anodyne use of 155
Cancer, on the use of arsenic in 31
———— account of an extraordinary (with a plate) 448
Cancerous affections, Mr. Rickards on 509
———— hydatids, observations on 193
Carbon, experiments to ascertain the quantity of it in the blood 192
Carcinoma, description of 193
Carlisle's, Mr. observations on the failure of union in simple fractures 201
———— remarks on ditto, by Mr. Wilkinson 397
———— Mr. his reply to Mr. Wilkinson 477
Carpue's, Mr. lectures announced 287
Cat, experiment on the chyle of a 200
Catalepsy, cases of, by Dr. Bouttatz 425
Cataract, on the use of Belladonna in the 352
Catarrhous complaints, the extractum cardui benedicti, recommended in 390
Caustic, on the use of, in ulcers 23

Page.
Chamberlaine, Mr. on the uses of the acoroides in complaints of the intestines 429
Chamomile, the juice of, recommended in scarlet fever 369
Chaussier's, Cit. method of preserving anatomical preparations 274
Chemical analysis of vaccine virus 395
Chemistry, on animal 416
Chenevix, Mr. on James's powder 517
Chevalier's, Mr. lectures announced 287, 289
Children, queries respecting those of the poor, by Dr. Beddoes 25
Chinese, their knowlege more antient than that of the Europeans 403
Chyle, how produced from chymus 209
Chymus and Chylus, on the nature of 193
Clarke's, Dr. letter to Dr. Bradley, on the cow pox 95
——— lectures announced 286
Clement, Mr. on vaccine inoculation 4
Clutterbuck's, Mr. case of erysipelas 13
Cold water, its use in cases of old ulcers 19
——— and ice, on the uses of 106
——— on the affusion of 381
Colour, enquiry concerning that of the blood 469
Conception, Mr. Hope's case of an extra-uterine 360
Consumption, review of Dr. Sutton's considerations on pulmonary 89
——— the seeds of phellandrium aquaticum recommended for 95
——— on the climate of Madeira, as beneficial in 415
Convulsions, on those which affect pregnant women 280
Coquart, Cit. on the effects of lightning and electricity in gonorrhœa 274
Cork, on the analysis of 276
Correspondents, answers to 96, 192, 380, 476
Cortex Caribœus, use of in rheumatic complaints 376
Cow-pox, Dr. Winterbottom's observations on 46
——— Dr. Waterhouse on 64
——— Mr. Jefferson's letter to Dr. Waterhouse on 69
——— Dr. Jenner's letter on inoculating dogs with 95
——— Dr. Clarke's letter to Dr. Bradley on *ib.*
——— Lord Berkeley's letter to Dr. Jenner on 105
——— Mr. Blount on 114
——— Mr. Dennett on 237

Page.
Cow-pox, Dr. Osiander's treatise on reviewed 253
——— in the sixth century 379
——— Mr. Ring's treatise on, announced 476
Cox's, Mr. account of a singular affection of the eyelids 393
Coxe, Dr. on the opium obtained from lettuce 396
Creaser, Mr. on spurious vaccine 326
——— chemical analysis of vaccine virus by 394
Crichton's, Dr. case of œdema fugax 26
——— cases of an undescribed cause of jaundice 29
——— observations thereon 312, 315
——— lectures announced 285
Crowfoot's, Mr. statement of a case of apoplexy 549
——— answer to the same 552
Cruickshank's, Mr. theory of galvanism 212
Cumming, Mr. on old ulcers 156
Currie, Dr. on the affusion of cold water 381
——— Mr. on the causes of the insalubrity of flat and marshy situations 493
Cutaneous diseases, description and treatment of, by Dr. Willan 297
Cuvier's, Cit. observations on the organ of the voice in birds 279
Davy's, Mr. eudiometer, description of 10
——— experiments on galvanism 214
Demanet, Cit. on the convulsions of pregnant women 280
Dennett, Mr. on the cow-pox 237
Dennison's, Dr. lectures announced 286
Desparanges, Cit. on the effects of acetic ether in rheumatism and the gout 273
Diet, review of Dr. Nisbet's practical treatise on 173
Digitalis purpurea, on the use of, by Mr. Simmons 133
——— the use of in hernia 322
——— in the dropsy 418
Discoveries, a sketch concerning new 407
Diseases in the Bath dispensary, state of 195, 364, 388, 491
——— in the Bristol dispensary 4, 112, 196, 364, 389, 491
——— in an eastern district of London, from the 20th May to the 20th June, 1801 8, 9

Page.

Diseases, account of, in the eastern district of London, from the 20th June to 20th July 111
——— from the 20th July to the 20th August 197
——— from the 20th August to the 20th September 296
——— from the 20th September to the 20th October 389
——— from the 20th October to the 20th November 490
——— in the Westminster hospital, from the 27th April to the 24th June, 1801 9
——— from 24th June to 24th Auguft 198
——— from August 24th to October 26th 391
Dissection, Mr. Syer's case of 507
Dissections, review of Mr. C. Bell's system of 80
Dogs, Dr. Jenner on inoculating 95
——— on the distemper of 315
——— Additions to that paper 466
Dolores post partum, relieved by the oil of sweet almonds 71
Dolor Faciei, on the history and nature of the 153, 243
Dropsy, on the use of the digitalis in 418
Drowned persons, on the wind-pipe in 93
Dyson, Mr. on vaccina 219
Earwax, analysis of the 475
Edinburgh, account of the medical education in the University of 301
——— Statutes of that university on medical degrees 306
Electricity, on the effects of, in gonorrhœa 274
Elongation of the tongue, Cit. Lassus on a morbific 353, 435, 522
Emetics, in pulmonary consumption, recommended 90
Endemic, disease in Norway, described, 18
———Ophthalmia of Ægypt, nature of 357
Epilepsy, observations on 377
———Cases of, by Dr. Bouttatz 425
Erysipelas, Mr. Clutterbuck's case of 131
Essays, announcement of Dr. Beddoes's popular 362
Eudiomiter, description of Mr. Davy's 10
Eudiometry, observations on 376
Experiments on phosphoric acid 92
——— on the Carbon in the blood 192
——— on land and sea air, 203, 359

Page.

Experiments, on galvanism 214
——— on the mesembryanthemum crystallinum 371
——— on the muriatic acid 475
Extra-uterine conception, case of 360
Extractum, cardui benedicti the uses of in catarrhous complaints 370
Eye, account of an extraordinary tumour in the (with plates) 289
Eye-lids, a singular affection of the 393
Fabæ pechurim, the efficacy of 376
——— botanical description of 377
Family physician, Review of Dr. Thomson's 176
Fawssett, Dr. on spurious vaccine 117
Febrile diseases, Review of Dr. Wilson's treatise on 255, 365
Femur, case of lacerated 127
Ferguson, Mr. on the use of cold water in old ulcers 19
Fermor, Mr. on vaccine inoculation 323
——— Dr. Jenner's letter to 325
Fever, on the use of vinegar in 107
——— on the affusion of cold water in *ib.*
——— Dr. Currie, on the affusion of cold water in 381
Fever, Review of Dr. Reich's treatise on 86
Fever-powder, analysis of Dr. Hahnemann's 475
Flachsland, Dr. case of monstrosity 94
Flat and marshy situations, on the insalubrity of 493
Fletcher, Dr. on a cause of jaundice 312
Forbes, Mr. on the distemper of dogs 314, 466
Fossils, on the water contained in 375
Fossil, analysation of, by Cit. Vauquelin 570
Fourcroy, Cit. his examination of the colouring part of the blood 469
——— on the urinary stones and gravel in men 527
Fracture, cases of by Mr. Carlisle, 201
——— remarks on ditto. by Mr. Wilkinson 397
——— case of, by Mr. Syer 505
——— Mr. Carlisle's reply to Mr. Wilkinson on 477
Gadolinit, Cit. Vauquelin, on the 188
Galvanism, on the progress of 209
——— experiments on 214
Gapper, Mr. on the external use of laudanum in hiccup 17
Garnett, Dr. his lectures announced 192
Geneva, the progress of vaccine inoculation at 281
Geohegan, Mr. on the venereal disease, review of 130

Page.
Goettling, Prof. his analysis of Dr. Hahnemann's fever-powder 475
Gonorrhœa, review of Mr. Geohegan's observations on 180
——— on the effects of lightning and electricity in 277
Goodwin's Mr. case of angina pectoris 320
Gout, the effects of acetic ether in the 273
Gravel in men, observations on by Cit. Fourcroy 527
Greece, practice of midwifery in 151
Grose, Mr. on hæmorrhœa petechialis 233
Hahnemann's, Dr. remedy for the scarlet fever 367
——— fever-powder, analysis of 475
Handel's Dr. remedy for the toothach 189
Hauy's new work on mineralogy, announced 282
Hecker's, Prof. cases of angini polyposa, spasmadocia 94
Hemorrhœa, petechialis, Mr. Grose on 233
Hepatic gas, on the vapours of in arthritic pains 92
Herder, Dr. on the internal use of phosphoric acid 92
Herholdt, Mr. on respiration 462
Hernia, on the use of digitalis in 322
Hernia umbilicalis, Cit. Portal's, account of 278
Herz, Dr. Marcus against vaccine inoculation 571
Hiccup, on the external use of laudanum in 17
Hildebrand's Prof. pocket book for health, reviewed 175
Himley, Prof. on a paralysis of the pupil of the eye 276
Honey-stone, analysis of the 189
Hope, Mr. his case of extra-uterine conception 360
Hospitals, Mr. Blair's proposals for a history of 363
Howard, Mr. on digitatis in the dropsy 418
Hufeland, Prof. on the medical use of oil 69
——— his system of practical medicine reviewed 170
——— on naptha vitrioli &c. in rheumatic and arthritic pains 378
Hull's, Dr. observations on the phlegmatia dolens 51, 135, 220
Humby, Mr. on vaccine inoculation 45
Hutchinson's, Mr. case of lacerated femur 127

Page.
Hydatids, Mr. Blair on cancerous 193
Hydrocephalus, Dr. Schmitt's case of 6
Hydrophobia, on the use of belladonna in 93
Ice, Dr. Rogers on the use of 106
Impregnation, review of Mr. Pulley on animal 254
Indigenous Corn, on the nature and generation of the earthy particles in different kinds of 568
Inoculated cow-pock. Dr. Winterbottom's observations on 46
Inoculation, vaccine Mr. Clement on 4
——— Dr. Paterson on 36
——— Mr. Humby on 45
——— Dr. Jenner on 95, 325
——— Dr. Clarké on 95
——— Address to the poor on 115
——— Mr. Fermor on 323
——— London testimonial in favour of 239
——— Progress of in Geneva 281
——— Dr. Stokes on 386
——— Proposal on the discovery of 423
——— Mr. Ring's remarks on 486
——— Mr. Phythian's cases of 489
Insalubrity of flat and marshy situations, on the causes of 493
Intelligence, medical and physical 568
Isenflamm, Prof. on the marrow of the bones 470
Jackson's Dr. elements of clinical practice announced 380
James's, Dr. powder, Mr. Chenevix on 518
Jaundice, an undescribed cause of a, by Dr. Crichton 29
——— observations on that case 218, 312, 313
Jefferson's, Mr. president letter to Dr. Waterhouse on the cow-pox 69
Jenkinson, Mr. on opiate frictions 432
Jenner, Dr. notice of Mr. Smith's print of 96
——— on inoculating dogs 95
——— letter to from Lord Berkeley 105
——— his reply to Mr. Fermor 325
Karsten, Mr. on the analysis of cork &c. 276
Kilburn-waters, Mr. Vandier's observations on 240, 513
——— Mr. Bliss's observations on 332
Kilian's, Dr. genius of health and life reviewed 253
Killer, Mr. on the use of digitalis in hernia 322
Kine-pock, Dr. Waterhouse on the 64

Page.
Kinglake, Dr. on the cure of arthritic 454
——— his question to Dr. Bouttatz 461
Kobes, Mr. on the fabæ pichurim 377
Koenig, Mr. on the practice of midwifery in Greece 151
Kruger's, Mr. method of preparing concentrated vinegar 474
——— method of preparing the nervous tincture *ib.*
Lampadius, Prof. on the water contained in fossils 375
Land and sea air, experiments on 203, 359
Lassus, Cit. on the hernia umbilicalis 278
——— on the elongation of the tongue 353, 435, 522
Laudanum, on the external use of in hiccup 17
LECTURES ANNOUNCED,
Dr. Garnett's on the theory and practice of medicine, on the animal economy, on chemistry and natural philosophy, 192. at St. Thomas and Guy's hospitals, 284. at St Bartholomew's hospital, *ib.* at the London hospital, 285. at the university of Glawsgow *ib.* at St. Mary-le-bone infirmary *ib.* Dr. Crichton's on medicine and chemiftry, *ib.* Dr. Bradley's on the theory and practice of medicine and chemistry, *ib.* and 380. Dr. Osborn's and Dr. Clarke's on the principles and practice of midwifery, and the diseases of women and children, 286. Dr. Batty's on midwifery and the diseases of Women and children, *ib.* and 380. Dr. Dennison and Dr. Squire on the same, *ib.* Mr. Pearson on the principles and practice of surgery, *ib.* Mr Pole on the theory and practice of midwifery &c. *ib.* Mr. Chevalier on the principles and operations of surgery, 287, 380. Mr. Thomas on the principles and practice of surgery, *ib.* Mr. Wilson on anatomy, *ib.* Mr. Carpue on anatomy, *ib.*
Lefebure, Prof. his researches on the nervous fluid reviewed 175
Leg, artificial, Mr. Potts on 445
——— Mr. Sheldrake on 447
——— Mr. Reid on 547
Lepra Grecorum, Mr. Pears's case of 124
Lettuce, on the opium extracted from 396
Light, its influence on phosphorus 374
Lightning and electricity, their effects in gonorrhœa 277
Literature, medical and physical, critical retrospect of, see *Books*

Page.
Living animals, on the odours exhaled by 339, 439
Locked-jaw, on the use of opium against the 280
London, testimonial in favour of vaccine inoculation 239
Madeira, Dr. Adams on sending consumptive patients to 415
Marius, Bp. of Lausanne in the 6th. century acquainted with the cow-pox 379
Marrow, observations on the motion of the spinal 370
——— of the bones, observations on 470
Marshy situations, causes of the insalubrity of 493
Martial flowers, mode of preparing 468
Medical Society, a print of, proposed 96
——— Benevolent, resolutions of the 113
——— pharmacopœia, review of 83
——— lectures, see *lectures*
——— practitioners, on the utility of an annual list of 334, 438
——— and physical intelligence 91, 185, 272, 367, 467
Medical and physical Literature, foreign and domestic critical retrospect of 77, 170, 253, 365, 563
Medicina Nautica, 3d. volume of announced 282
Medley, Mr. his proposals for a print of the medical society 96
Membranes, Cit. Bichat's observations on the 448
Mental derangement, singular case of 277
Mercurial gout, description of 92
Mesen branthemum crystallinum, Dr. Wendt on 371
Metallic brush, description of 91
Metallic bodies, change of by lava 570
Meteorological Register kept at Cambridge in North America, from June 23, to July 16, 1801. 321
Michaelis, Dr. G P. on the most proper arrangement of Field Hospitals 563
Midwifery in Greece, account of 151
Mineralogy, Mr. Hauy's new work on, announced 282
Minerals, method of analyfing 373
Moffatt, Mr. on animal chemistry 416
Molwytz's metallic brush, account of (with a plate) 91
——— on the vapours of the hepatic gas 92

Page.
Monstrosity, a singular instance of 94
Motion in the spinal marrow, observations on 370
Muller's History of Switzerland quoted 379
Munk, Dr. on an endemic disease in Norway 13
Muriatic acid, experiments on the 475
——— ether, method of preparing 16
Mursinna, Mr. on the cure of tetanus 280
Naptha Vitrioli, Prof. Hufeland's experiments with 378
Nervous tincture, method of preparing Bestucheff's 474
Nisbet's, Dr. practical treatise on diet reviewed 178
Noef, Mr. on the juice of Plantago angustifolia 276
Norway, account of an endemic disease peculiar to 13
Odours, on those exhaled by living animals 339, 439
Oedema fugax, Dr. Crichton's case of 25
——— Dr. Yelloley's case of 168
Oesophagotomy, Mr. Blair on (with a plate) 97
Oil, on the medical use of 69
—— of sweet almonds, efficacious in dolores post partum 71
—— on the efficacy of olive 247
Opiate friction, Mr. White on 387
——— Mr. Ward on 431
——— Mr. Jenkinson on 432
——— Mr. Boutflower on 433
Opium, on its use against the tetanus 280
——— Dr. Coxe, on that extracted from lettuce 396
Opium, Mr. Ward's observations on the external application of 478
Ophthalmia, Dr. Savaresi's account of the Ægyptian 357
Ornithorhynchas paradoxus, anatomical remarks on (with a plate) 73
Osiander, Dr. his treatise on the cow-pox reviewed 253
Paget, Mr. on belladonna in cataract 352
——— on an extraordinary calculus (with an engraving) 391
Palermo, success of the cow pox at 95
Pananach well, description of 19
Paralysis, cases of by Mr. Purton 1
——— of the pupil of the eye by extractum hyosciami 276
Paris, establishment for mineral waters at 185
Paterson, Dr. on vaccine inoculation 36
———'s, Dr. case of uncommon tumour 309
——— on new discoveries 406

Page.
Paul and Co's establishment for the preparation of mineral waters at Paris 185
Pears's, Mr. case of lepra grecorum 124
Pearson's, Dr. letter to Mr. Thomas on an abscess 295
Peck, Mr. on caustic in ulcers 19
——— on quackery 129
Perron's, Cit. observations on the uterus 278
Pharmaceutical processes 15
Phellandrium aquaticum, use of the seeds of 95
Philo-medicus on an annual list of regular medical practitioners 334, 438
Phlegmatic dolens, observations on, by Dr. Hull. 51, 135, 220
Phosphate of lime, how procured 520
Phosphoric acid, on the internal use of 92
Phosphorus the influence of light upon 374
——— Prof. Hufeland's experiment on 378
Phythian's, Mr. cases of vaccine inoculation 489
Plague, on the efficacy of olive oil in preventing the 247
——— description of the 259, 365
Plantago angustifolia, on the Juice of 276
Ploucquet, Prof. on the wind-pipe in drowned persons 93
Poor, address to the, on vaccine inoculation 115
——— queries relating to the children of the 25
Portal's, Cit. observations of a motion in the spinal marrow 370
——— on epilepsy 377
Posewitz, Dr. his journal for medicine reviewed 79
Potts Mr. on artificial legs 445
——— Mr. Reid's letter to 547
Powder, analysis of Dr. Hahnemann's fever 475
Mr. Chenevix, on Dr. James's 518
Pox, case of small 3
Pregnant women, on convulsions in 279
Primrose's, Mr. case of mortified uterus 331
Prunus laurocerasus, on the distilled water of 375
Pulley's Mr. essay on animal impregnation reviewed 254
Pulmonary consumptions, Dr. Sutton's treatise on reviewed 89
Purton's Mr. cases of paralysis 1
Quackery, Mr. Peck's remarks on 129
Queries, on the condition of the children of the poor, by Dr. Beddoes 25

Page.
Question, from Dr. Kinglake to Dr. Bouttatz 461
Quicksilver, method of preparing the syrup of 15
Radesyge, account of an endemic disease in Norway, so named 13
Reece's, Mr. medical and chirurgical pharmacopœia, reviewed 88
——— on an instrument for extracting teeth 157
Reich, Dr. on fever, translated by Mr. Parry, reviewed 86
Reid's, Mr. letter to Mr. Potts 547
Resolutions of the benevolent medical society, Essex and Herts 113
Respiration, of the power of the will on 372
——— observations on, by Mr. Herholdt 462
Resuscitative apparatus, description of a 84
Rheumatism, effects of acetic ether in the 273
Rhus Rhadicans, account of 272
Rickards, Mr. on cancerous affections 509
Rickets, remarks on the cause of 191
Ring, Mr. on the anodyne virtue of camphor 155
——— his treatise on the cow-pox announced 476
——— his remarks on vaccine inoculation 486
Ritter, Dr. on the use of snails in obstinate buboes 93
Rogers, Dr. on the use of cold water and ice 106
Rolfe's, Mr case of small-pox 3
——— on yeast in typhus 112
Roose, Prof. on the power of the will on respiration 372
Rule, Mr. on the prevention of bilious fever 311
Savaresi, Dr. on the endemic ophthalmia of Ægypt 357
Sauter, Dr. on the use of belladonna in hydrophobia 93
Saxon beryll, analysis of the 473
Scarlet fever, Dr. Hahnemann's remedy for 367
——— Mr. Skrimshire on 420
Scheel, Dr. on the efficacy of olive oil 247
Schmidt's, Mr. method of preparing the Æthiop's antimonialis 15
Schmitt's, Dr. case of hydrocephalus 6
Schumacher, Mr. on the faba pichurim 376
——— on the cortex caribæus *ib.*
Scirrhous Pancreas, considered as a cause of jaundice 29, 218, 312, 313

Page.
Selig, Dr. on the uses of extractum cardui benedicti in catarrhous complaints 3
Semina Phellandrii Aquatici, on the uses of 95
Seybert's, Dr. experiments on land and sea air 203, 359
Sheldrake, Mr. on artificial legs 447
Sicily, the cow-pox greatly encouraged in 95
Siebold, Prof. on the dolor faciei 158, 243
Simmons, Mr. on the use of arsenic in cancer 31
——— on digitalis 133
Skrimshire, Mr. on scarlet fever 420
Small-pox, Mr Rolfe's case of 3
Smith, Mr. his print of Dr. Jenner noticed 96
——— W. on an inversion of the uterus 503
Snails, on the use of, in obstinate buboes and scrophulous ulcers 95
Spinal marrow, observation of a motion in the 370
Statutes of the university of Edinburgh on medical degrees 306
Stern, Mr. on semina phellandrii aquatici 95
Stevenson, Mr. on spurious vaccine 121
Stokes, Dr. on vaccine inoculation 386
Struve's, Dr. essay on suspended animation, reviewed 83
Suspended animation, essay on, by Mr. Bishop 315
Sutton's, Dr. considerations on pulmonary consumption reviewed 89
Syer's, Mr. case of fracture 505
——— dissection 507
Syrup of quicksilver, method of preparing 15
Tartarous acid, on the transition of 475
Tatham's new-invented truss, account of 282
Teeth, account of an instrument for extracting 157
——— Mr. Custance on extracting 251
Tetanus, on the use of opium as a remedy for 280
Thomson's, Dr. family physician, reviewed 176
Tobacco, in cases of suspended animation exploded 118
Tongue, on the morbific elongation of the 353, 435, 522
Tooth-ach, Dr. Handel's remedy for the 189
Toxciodendron, botanical description of 272
Tromsdorff's, Prof. method of preparing the muriated barytes without kali 467

Page.
Tromsdorff's, Prof. analysis of the Saxon beryll 473
Trotter's, Dr. announcement of a 3d vol. of his Medicina Nautica 282
Tumor, account of an extraordinary one in the eye (with plates) 289
——— Dr. Patterson's case of an uncommon 309
Typhus, on the use of vinegar in 107
——— on the use of yeast in 112
Ulcers, on the use of cold water in old 19
——— on the use of caustic in 24
——— scrophulous, cured by the application of snails 92
——— Mr. Cumming on old 156
Urinary stones and gravel, in men, Cit. Fourcroy on the 527
Urine, on the use of blisters in suppression of the 526
Uterus, Cit. Penou's observations on 278
——— case of mortified 331
——— Mr. Smith's case of an inversion of the 503
Vaccine, Mr. Dyson on 219
——— Dr. Barry on 423
——— Dr. Fawssett on spurious 117
——— Mr. Stevenson on ditto 121
——— Nole on ditto 218
——— Mr. Creaser on ditto 326
——— Inoculation, Mr. Clement on 4
——— Dr. Paterson on 36
——— Mr. Humby on 45
——— address to the poor on 115
——— London testimonial in favour of 239
——— progress of, at Geneva 281
——— Mr. Fermor on 323
——— Dr Jenner on 95, 325
——— Dr. Stokes on 386
——— proposal on the discovery of 422
——— Mr. Phythians cases of 489
——— Mr. Ring's remarks on 486
Vaccine virus, Dr. Waterhouse on 327
——— chemical analysis of 395
Vandier, Mr. on the Kilburn waters 240, 513
——— on the vitality of the blood 513
Van Mons, Cit. his method of preparing muriatic ether 16
——— on the Rhus Rhadicans 272
Vaquelin's, Cit. account and analysis of the Gadolinit 188
——— analysis of the honey-stone 189

Page.
Vaquelin's, Cit. method of analysing minerals 373
——— examination of the colouring part of the blood 469
——— analysis of the ear-wax 475
Van Stichell, Dr. on cachexy 376
Vapours of hepatic gas, Mr. Molwitz on the 92
Venereal disease, Mr. Geohegan's practical observations on, reviewed 180
Vinegar, beneficial in typhus 107
——— method of preparing the concentrated 474
Virey, Mr. on the odours exhaled by living animals 339, 439
Vitality of the blood, on the 513
Voice in birds, on the organ of 279
Ward, Mr. on opiate friction 431
Ward, his observations on the external application of Opium 478
Water cold, use of in old ulcers 19
——— affusion of, in fever 381
——— Dr. Rogers on the use of 106
——— contained in fossils, Prof. Lampadius on 375
——— distilled of prunus laurocerasus, on 375
Waterhouse, Dr. on the kine-pock 64
——— Mr. Jefferson's letter to 69
——— on vaccine virus in hot weather 327
Wax, analysis of the ear 475
Weather, on the vaccine virus in hot 327
Wendt, Dr his account of the mesembryanthemum crystallinum 371
Westminster hospital, *v. Diseases in the Westminster hospital.*
White, Mr. on opiate friction 387
Whitford, Mr. account of his newly invented truss 283
Wiedemann's archives for zoology and zootomy, reviewed 79
Wilkinson, Mr. on the union of simple fracture 397
——— Mr. Carlisle's reply to his remarks 477
Will, on the power of the, on respiration 372
Willan, Dr. on cutaneous diseases 297
Wilson, Dr. on febrile diseases, reviewed 255, 365
Windpipe in drowned persons, Prof. Ploucquet on the 93
Winterbottom's, Dr. observations on the inoculated cow-pock 6
Women, on the convulsions peculiar to pregnant 279

Page.

Wood's, Mr. case of Cæsarean operation (with a plate) 346

Wrisberg's, Mr. medical treatises reviewed 77

Wurzer, Prof. on the distilled water of prunus laurocerasus 375

Yeast, its efficacy in typhus, by Mr. Rolfe 112

Yelloley, Dr. his case of oedema fugax 168

Zoology and Zootomy, Prof. Wiedeman's archives for, reviewed 79

REFERENCES TO THE PLATES.

Skull of the Ornithorhynchus Parodoxus, &c. - - - Page 73

Hemispherical Acoustic Tube, by Professor Arneman - - - 73

The Metalic Brush, - - - - - - - - 73

Lepra Grecorum, or Psoriasis Inveterata - - - - 124

Mr. Blair's Operation of Œsophagotomy - - - - - 97

Dr Bouttatz's Case of an extraordinary Tumour of the Eye, Plate 1 & 2 289, 293

Mr. Paget's extraordinary Case of Calculus - - - 393

Cancer on the Cheek (Case of Mr. Atkinson) - - - - 449

Case of Cæsarian Operation (Mr. Wood's) - - - - 449

NEW MEDICAL BOOKS, juſt publiſhed by R. PHILLIPS,

No. 71, St. Paul's Church-yard.

I. In one volume, duodecimo, price 6s. in boards, A PRACTICAL TREATISE ON DIET, and on the moſt ſalutary and agreeable means of ſupporting Life and Health by Aliment and Regimen.—Adapted to the various circumſtances of age, conſtitution, and climate, and including the application of modern Chemiſtry to the culinary preparation of Food. By William Nisbet, M. D. Fellow of the Royal College of Surgeons, Edinburgh ; author of the Clinical Guide.

II. Illuſtrated by a coloured plate, repreſenting the Puſtules in five different ſtages ; a ſecond edition, price 2s. 6d. A conciſe View of the moſt important Facts which have hitherto appeared concerning THE INOCULATION FOR THE COW POX. By C Aiken, Member of the Royal College of Surgeons.

III. In one thick volume, duodecimo, price 5s. in boards, or 6s. bound, containing more letter-preſs than any other medical work of a general and popular nature, THE FAMILY PHYSICIAN, or DOMESTIC MEDICAL FRIEND : Containing plain and practical Inſtructions for the Prevention and Cure of Diſeaſes, according to the laſt Improvements and Diſcoveries, with a Series of Chapters on collateral Subjects ; compriſing every thing relative to the Theory and Principles of the medical Art neceſſary to be known by the private Practitioner ; the Whole diveſted of ſcientific and technical Words and Phraſes, and adapted to the Uſe of thoſe Heads of Families who have not had a claſſical or medical Education. By Alexander Thomson, M. D. Author of Medical Conſultations ; of an Eſſay on the Diſeaſes of Women ; of a Treatiſe on the Nerves, &c. &c. &c.

IV. In one volume, duocecimo, price 5s. in boards, REPORTS ON THE DISEASES IN LONDON, during the Years 1796, 1797, 1798, 1799, and 180o, with practical Obſervations, &c. &c. &c. By Robert Willan, M. D. F. A. S.

T. Gillet, Printer, Salisbury-square.

